# The non-attached biofilm aggregate

**DOI:** 10.1038/s42003-023-05281-4

**Published:** 2023-09-01

**Authors:** Kasper N. Kragh, Tim Tolker-Nielsen, Mads Lichtenberg

**Affiliations:** Costerton Biofilm Center, Department of Immunology and Microbiology, University of Copenhagen, Copenhagen, Denmark

**Keywords:** Biofilms, Bacteriology

## Abstract

Biofilms have conventionally been perceived as dense bacterial masses on surfaces, following the five-step model of development. Initial biofilm research focused on surface-attached formations, but detached aggregates have received increasing attention in the past decade due to their pivotal role in chronic infections. Understanding their nature sparked fervent discussions in biofilm conferences and scientific literature. This review consolidates current insights on non-attached aggregates, offering examples of their occurrence in nature and diseases. We discuss their formation and dispersion mechanisms, resilience to antibiotics and immune-responses, drawing parallels to surface-attached biofilms. Moreover, we outline available in vitro models for studying non-attached aggregates.

## Abstract (web summary)

This review discusses the biology of non-attached biofilm aggregates, including the mechanisms by which they are formed and available in vitro models for studying their features.

## Introduction

The surface attached biofilm, as drawn by Stoodley et al.^1^ and reprinted countless times in theses, reports, and journal articles ever since, is well studied due to its conspicuous nature, ease of handling and phenotype that is in stark contrast to the planktonic lifestyle. Non-attached aggregates have not received the same attention although they have regularly been described in the literature. For example, Pasteur described how floating aggregated bacteria could spoil wine production in 1864^2^. And aggregated bacteria have been described in freshwater and marine environments for close to a century^3,4^. In 1977, Høiby et al. described so-called heaps of bacteria surrounded by inflammation in chronic pneumonia patients with cystic fibrosis (CF)^5^, and the following year Bill Costerton published his defining biofilm theory^6^. In the following decades surface attached biofilms were the focus of numerous studies, and it is only in the last decade that the biology of non-attached aggregates has been intensively studied. Cai recently contributed a review describing non-attached microbial aggregates with focus mostly on microbial ecology aspects^7^. Here we review our current knowledge of non-attached aggregates focusing on molecular- and microenvironmental aspects with an emphasis on aggregates found in human disease.

### Non-attached aggregates are prevalent in nature and disease

In the laboratory, experimental microbiologists will occasionally observe macroscopically visible aggregates in liquid batch cultures. In addition to the visible aggregates, Schleheck et al. described that a considerable part of the biomass in a *P. aeruginosa* batch culture is bound in microscopic, suspended aggregates ranging from 10 – 400 µm in diameter^8^, and not only confined to the air-liquid interface as e.g., pellicles^9^. Moreover, *Staphylococcus aureus* has been reported to start aggregating in the early exponential phase of growth in batch cultures, resulting in more than 90% of the population being bound in aggregates when they reach the stationary phase^10^. Such aggregates can have a profound impact on e.g., growth rate and antibiotic tolerance in in vitro experiments^11,12^.

Flemming et al. compiled a meta-analysis on the occurrence of biofilms in different habitats and reported the presence of aggregates in many environmental habitats^13^. A prominent example of non-attached aggregates in nature is the pelagic aggregates, often referred to as marine- or lake snow. They have been widely described as a mode for bacteria to colonize the open water column^14,15^. These aggregates are composed of microbial communities held together by self-produced extracellular polysaccharides or as part of inorganic and organic particles such as clay or sand minerals, fecal pellets, or organic debris. The free-floating aggregated phenotype in limnic and marine environments may confer many of the same attributes to bacteria as often ascribed to classical surface biofilm. E.g., bacteria can attain protection against grazing and live in a stable chemical environment. Further, bacteria bound in pelagic aggregates have been shown to contain elevated amounts of several quorum-sensing related molecules such as N-acyl homoserine lactones compared to their single-celled counterparts^16,17^. The size of the pelagic aggregates is typically in the range of 500 µm to centimeters.

**Fig. 1:**
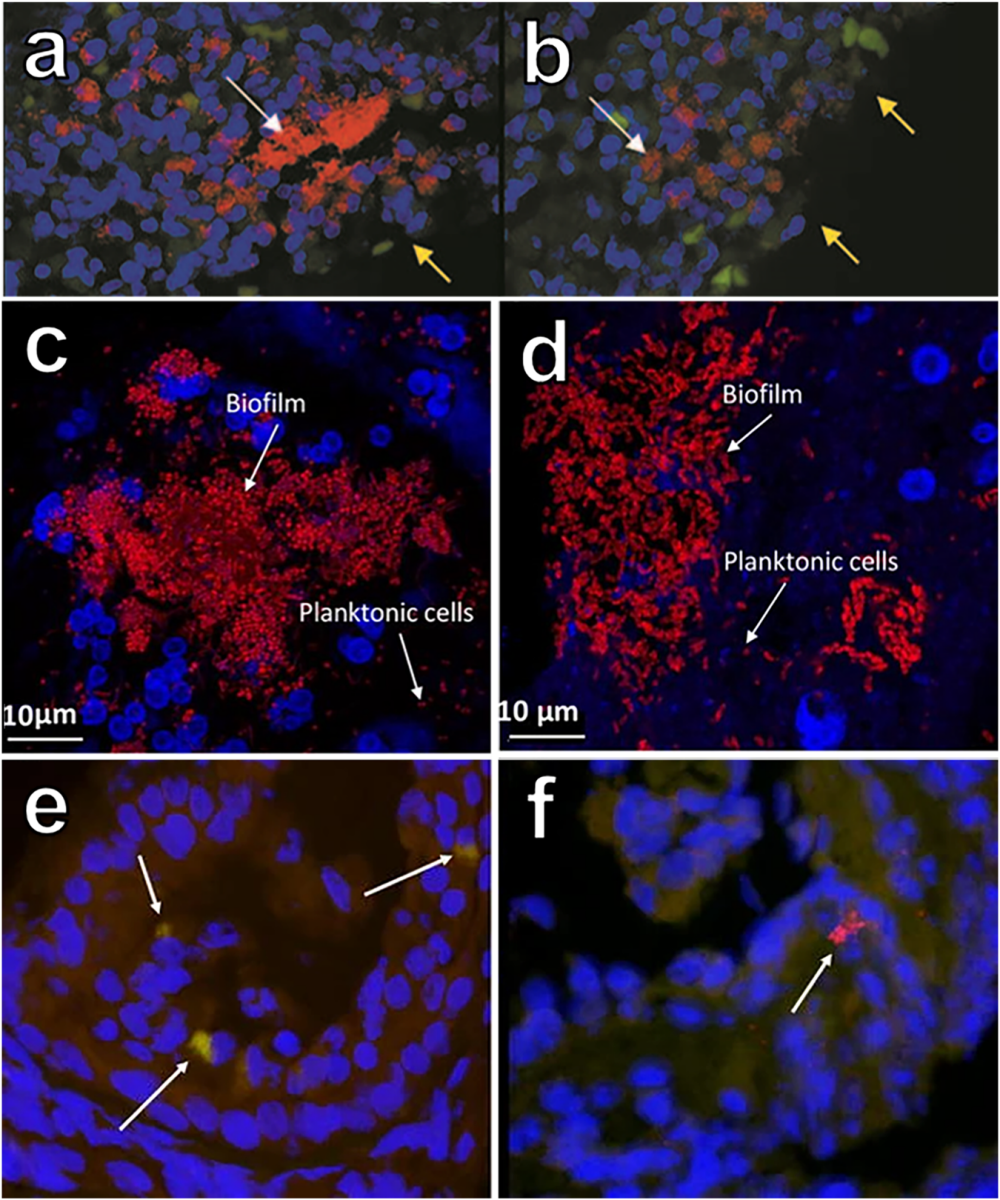
Examples of non-attached aggregates found in chronic infections. **a**, **b** Biofilm aggregates in debridement material from chronic wounds stained red with PNA-FISH probes. White arrows indicate biofilm aggregates, yellow arrows indicate the surface of the wound. Biofilm aggregates found in sputum from patients diagnosed with either (**c**) community acquired pneumonia or (**d**) cystic fibrosis. Red represents cells stained with bacteria specific PNA-FISH. **e**, **f** Specimens from chronic otitis media. **e** Aggregates of Staphylococcus aureus stained with specific PNA-FISH probes (green-yellow) and (**f**) aggregate stained with a universal bacteria PNA-FISH probe indicated with white arrows. In all panels, host cells were stained blue with DAPI. Adapted with permission from^21,22,79^.

**Fig. 2:**
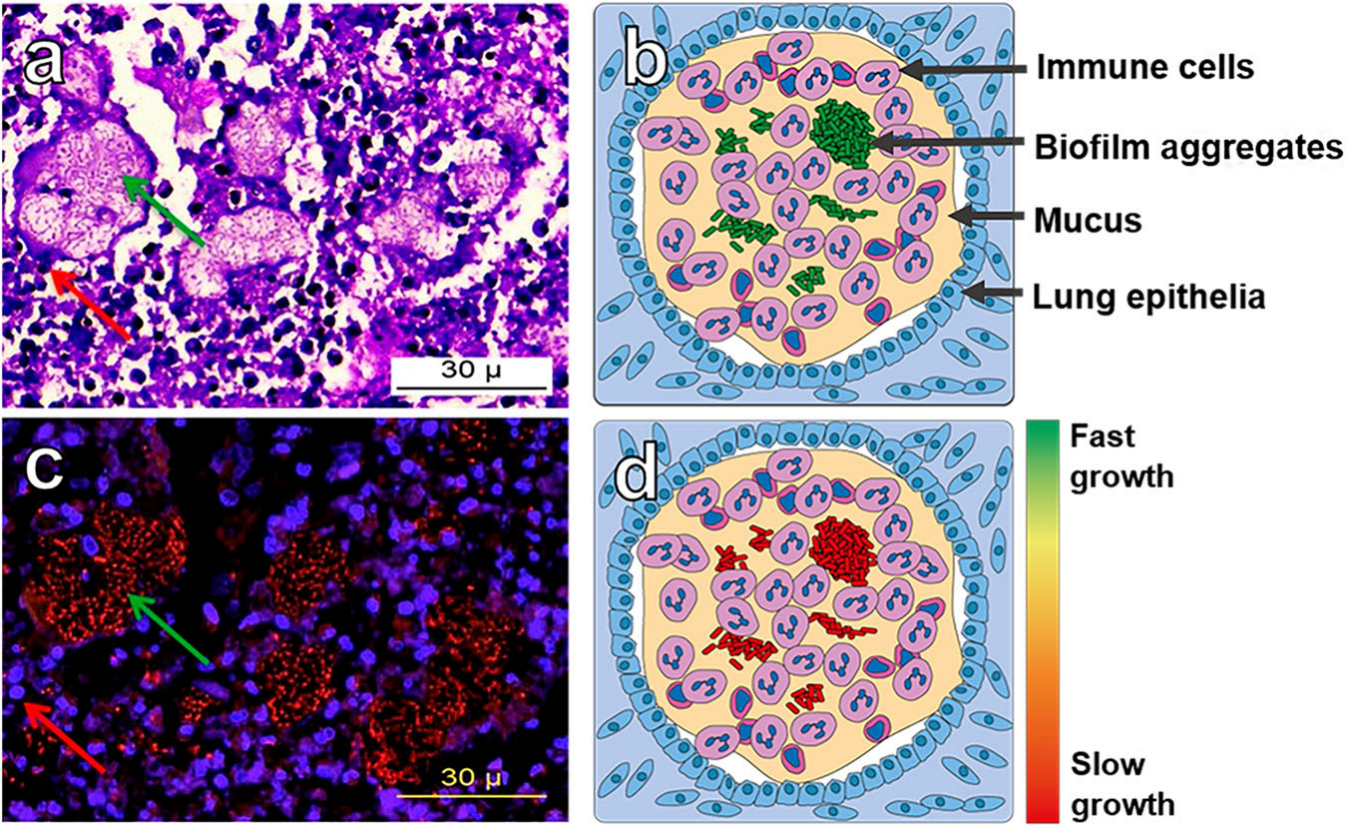
The infectious biofilm aggregate. **a** HE stained explanted lung tissue from a patient with cystic fibrosis. Image focused on a bronchial lumen. Green arrow indicates a non-attached bacterial aggregate, and the red arrow indicates a host immune cell associated with the aggregates. The unit of the scalebar refers to µm. **b** Schematic drawing of chronic biofilm infection in CF lungs. Bacteria grows as small non-attached aggregates (green cells), in obstructed bronchia filled with mucus surrounded by a multitude of innate immune cells (pink cells). The aggregates have been found to be unassociated with the epithelial lining (blue cells)^27^. **c** The neighboring slide of the image A, stained for P. aeruginosa with specific PNA-FISH probes and counterstained with blue DAPI. The green arrow indicate a red stained P. aeruginosa aggregate and red arrow shows the DAPI stained nucleus of an immune cell. The unit of the scalebar refers to µm. **d** aggregates of many infections have been found to grow slowly due to limited availability of oxygen due to high consumption from immune cells. **a**, **c** are adapted from^81^ with permission.

Microbial aggregates are also prevalent in infections and Bjarnsholt et al.^18^ compiled a meta-analysis on the distribution of aggregates in chronic infections, and their characteristic sizes. They found non-attached aggregates with a range of 5 to 200 µm in diameter to be dominant in most chronic infections, such as CF-related infections, chronic wounds, otitis media, and chronic osteomyelitis^18–21^ (Fig. 1). However, Kolpen et al. recently identified non-attached aggregates in expectorate from patients admitted to the hospital with acute pneumonia, suggesting that biofilm aggregates are not only limited to chronic infections^22^. The aggregates contained exopolysaccharides and exhibited similar sizes as aggregates found in chronic pneumonia infection in CF or COPD patients. Similar results were recently found in a meta-analysis that revealed the presence of biofilms in acute wounds^23^. The use of advanced microscopy techniques, such a confocal laser scanning microscopy and electron microscopy, have revealed the structure and positioning of bacteria in several chronic bacterial infections^24^. A common denominator for many of these is that the bacteria are found positioned in small aggregates surrounded by polymer-rich host secretions and large amounts of inflammatory cells^19,25,26^ (Fig. 2). These aggregates do not appear to be intimately associated with intact epithelia or other eukaryotic surfaces^27^.

### Mechanisms of aggregate formation

**Fig. 3:**
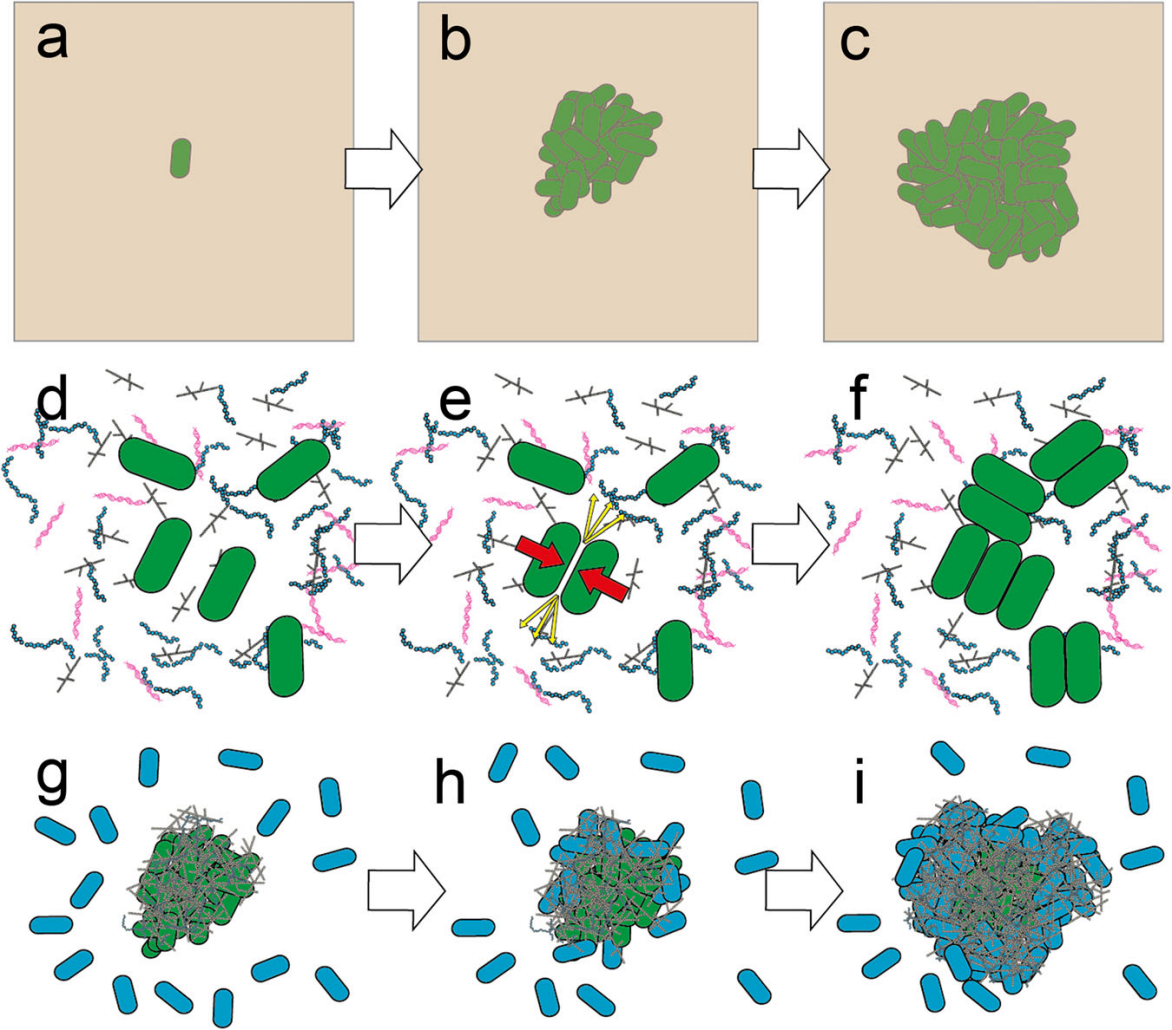
Schematic overview of three different proposed mechanisms behind aggregate formation. **a**–**c** Aggregate formation due to immobilization in viscous materials. **a** Single cells are immobilized in a viscous gel. **b** Over time the single cell proliferates to an immobilized microaggregate. **c** As the aggregate attains a certain size, its expansion becomes limited due to space restriction and/or resource stratification. **d**–**f** Depletion aggregation in polymer-rich environments. **d** Single bacterial cells found in polymer rich environment. **e** Cells in proximity of each other will attract due to entropy generated by osmotic imbalance (red arrows) as polymers move out from between the cells (blue arrows). **f** Depletion aggregation creates the distinct fence pattern aggregate formation. **g**–**i** Bridging aggregation. **g** Preformed aggregates (green cells) are in a biofilm state where they produce extracellular polymers. **h** Single cells (or other aggregates) are recruited to the aggregate, facilitated by the sticky polymers around the aggregates. **i** Over time the aggregates increase in size through growth, continuous recruitment, and incorporation of single cells and/or other aggregates.

Microbial aggregates are known to be formed by three different mechanisms: (1) due to restricted motility in high-density gels, (2) by depletion aggregation in polymer-rich environments, and (3) by bridging aggregation caused by bacterial extracellular polymers (Fig. 3).

Staudinger et al. demonstrated that *P. aeruginosa* can form aggregates in high density gels due to restricted motility^28^. In addition to wild type cells, aggregates were formed by mutants that were deficient in synthesis of exopolysaccharides and pili, indicating that aggregate formation does not require functions needed for development of surface associated biofilms. In addition, Pabst et al. demonstrated that *S. aureus* can form aggregates in an agar gel system^29^. These findings are highly relevant in the context of infections since high density conditions caused by polymer-rich host secretions are thought to be predominant at infectious sites^28^.

Secor et al. reported a series of in vitro experiments indicating that polymers that are abundant at chronic infection sites can cause *P. aeruginosa* cells to aggregate by a so-called depletion aggregation mechanism^30^. Depletion aggregation is mediated by entropic forces between like-charged polymers and the bacteria, and therefore it does not require biofilm formation functions such as self-produced exopolysaccharides.

Examples of the formation of microbial aggregates via bridging aggregation include a study by Kragh et al. who reported that *P. aeruginosa* aggregates need at least the two well-described exopolysaccharides Pel and Psl to maintain the coherence of aggregates in suspension^11^. In addition, the adhesin CdrA has been shown to be involved in the formation and maintenance of *P. aeruginosa* aggregates in liquid culture^31,32^.

In addition to the three described mechanisms of aggregate formation, it is possible that some non-attached aggregates can originate from surface attached biofilms due to shedding or break-off of a part of the biofilm.

### Role of the extracellular matrix of aggregates

The extracellular matrix of non-attached *P. aeruginosa* aggregates has been shown to be strikingly similar to that of surface attached biofilms^33^. In addition, self-assembled *Staphylococcus epidermidis* aggregates in liquid culture had increased expression of proteins related to extracellular matrix production, like polysaccharides and eDNA secretion^34^. Moreover, in vitro grown *S. aureus* aggregates were shown to contain substantial amounts of the polysaccharide intercellular adhesin (PIA), well-known for its involvement in the establishment of surface-bound biofilm^10^.

In suspension, the formation of *P. aeruginosa* aggregates has been linked to the active recruitment of surrounding single cells to a greater extent than clonal growth within the aggregates^11^. This recruitment process was meditated through the active secretion of especially Psl. It was speculated that Psl could act as an adhesin enabling the immobilization of planktonic cells on the outside of the aggregate.

Staudinger et al. demonstrated that bacteria can form aggregates independent of matrix components if they grow suspended in gel material that restrict their motility, and this process was suggested to play a role in the formation of aggregates at infectious sites^28^. Likewise, Secor et al. suggested that matrix-independent depletion aggregation plays a role in the formation of bacterial aggregates in chronic infections^30^. However, Jennings et al. provided evidence that depletion aggregation does not play a role for the formation of *P. aeruginosa* aggregates in the CF lung^35^. This was based on the fact that aggregates formed by depletion aggregation are organized differently than aggregates formed by bridging aggregation. The aggregates found in CF lung sputum had the organization typical of aggregates formed by bridging aggregation, indicating that their formation is mediated by the bacterial extracellular matrix^35^.

During the course of CF lung infection there is a strong selection for *P. aeruginosa* mutants that overproduce biofilm matrix components. Overproduction of alginate by *P. aeruginosa mucA* mutants enable the bacteria to develop persistent infections in the lungs of CF patients^5,36^. Overproduction of Psl and Pel by *P. aeruginosa* mutants such as *wspF* and *yfiR* also confer a benefit to the bacteria during CF lung infection^37–39^. Similarly, *P. aeruginosa wspF* mutants overproducing exopolysaccharide developed rapidly in a porcine burn wound model^40^. Overproduction of these polysaccharide biofilm matrix components may be important for the formation of aggregates at the infectious sites.

**Fig. 4:**
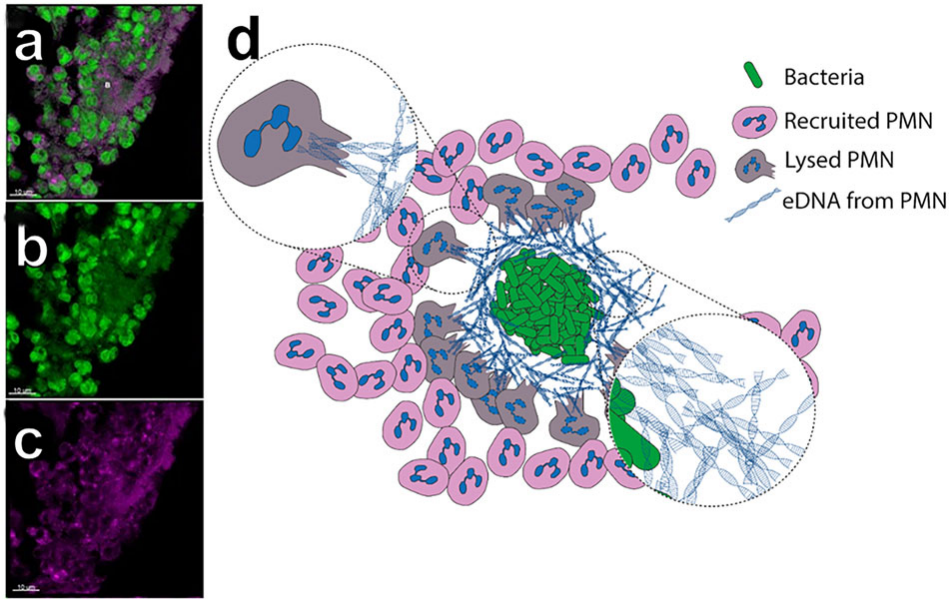
The origin of extracellular DNA surrounding biofilms. **a** Image of infected murine tissue. Both immune cells and bacteria can be seen as green cells. Eukaryotic DNA was stained purple with antibodies. **b** Murine immune cells can be seen as large green cells surrounding a large biofilm aggregate. **c** Antibody stained eDNA can be seen surrounding the biofilm aggregate. Scalebar in (**a**–**c**) is 10 µm. **d** Schematic drawing of the proposed mechanism behind the large amount of eukaryotic eDNA around biofilms. Leukocytes, in this case PMNs, are lysed by bacteria in the aggregates, resulting in expulsion of DNA from the lysed PMNs. Adapted from^45^ with permission.

Whitchurch et al. reported that eDNA functions as a matrix component in surface-associated *P. aeruginosa* biofilms grown in vitro^41^. Investigations of biofilm development in the presence of non-bacterial DNA suggested that *P. aeruginosa* can incorporate the foreign DNA as an integral part of the extracellular matrix^42–44^. Moreover, the presence of neutrophil immune cells was shown to stimulate *P. aeruginosa* biofilm formation through release of eDNA that was incorporated in the biofilms^44^. Alhede et al. showed that the majority of the eDNA surrounding the biofilm aggregates in murine infections and ex vivo lung tissue come from lysed host immune cells^45^ (Fig. 4). Under in vitro conditions the secretion of eDNA has been shown to be controlled by the Las quorum sensing system through the PQS system^46^. This Las regulation has primarily been shown in mature surface attached biofilms grown in vitro, so whether this regulation is operating in non-attached aggregates is not known.

Proteins can also play a role as extracellular matrix components of non-attached aggregates. For example, *Escherichia coli* is prone to aggregation mediated by the adhesin termed Antigen 43^47–49^. Ag43-dependent aggregates exhibit specific properties, including persistence and virulence of uropathogenic *E. coli* in the mouse bladder^48^.

Lastly, the host immunometabolism may influence the expression of EPS. For example, it was recently shown that *P. aeruginosa* and *S. aureus* can stimulate itaconate production by the host which can induce increased bacterial EPS production^50,51^. However, the current knowledge on this phenomenon is limited and whether the balance between non-attached vs. surface attached biofilm is affected by this is not known.

### Mechanisms of aggregate dispersal

As described above, aggregates have been shown to form through several active or passive mechanisms. Besides de novo formation of aggregates, non-attached aggregates can also originate by detaching from other aggregates or surface-attached biofilms. Hydrodynamic stress and shear forces can play a significant role in the detachment of bacterial aggregates from biofilms. Studies have shown that the strength of the biofilm matrix and the size and shape of the aggregates can affect their susceptibility to hydrodynamic stress^52,53^. In other instances, the presence of extracellular matrix-degrading enzymes can also contribute to detachment from surface-attached biofilm as it has been shown for *P. putida* during starvation^54^. The upstream detachment of aggregates can provide an advantage for subsequent downstream surface colonization as an aggregate may provide both instant protection and a growth advantage in the initial establishment steps compared to the single-cell attachment to a surface^12^.

There is some evidence for single-cell dispersal from non-attached aggregates upon starvation as well. In a study by Schleheck et al., it was found that *P. aeruginosa* preferentially grows as aggregates in liquid batch cultures^8^. However, upon carbon, nitrogen, or oxygen starvation, the floating aggregates dispersed into single cells^8^. These findings suggest that *P. aeruginosa* non-attached aggregates show similar behavior to what has been reported for *P. putida* surface-associated biofilms^54^. Interestingly, we observed a decrease in aggregated biomass fraction of *P. aeruginosa* in chemostatic cultures after 10 days but since these cultures are not limited in electron donors or -acceptors these results contradict the starvation induced dispersal^11^.

During the last decade it has become evident that many bacteria species employ c-di-GMP signaling to regulate biofilm formation^55^. Diguanylate cyclase enzymes catalyze formation of the c-di-GMP molecule, whereas c-di-GMP phosphodiesterase enzymes catalyze degradation of c-di-GMP in the bacteria. An elevated cellular level of c-di-GMP upregulates matrix production and drives planktonic bacteria to form biofilms, whereas a reduction in the c-di-GMP level induces dispersal of biofilm bacteria into the planktonic mode of life. In addition to their catalytic domains many of the diguanylate cyclases and phosphodiesterase enzymes have regulatory domains through which they are thought to regulate the bacterial life-style (planktonic versus biofilm) in response to environmental cues. A decrease in the level of c-di-GMP has been shown to result in dispersion of surface-attached biofilms in both in vitro systems and murine biofilm infection models^56–59^. However, it is not known whether this mechanism is relevant for dispersion of non-attached aggregates.

Quorum sensing has also been associated with regulation of biofilm dispersal. Changes in the acyl-homoserine lactone levels in *P. aeruginosa, Serratia marcescens* and *Vibrio vulnificus* have all been shown to induce dispersal of surface-attached biofilm^60–62^. However, it is not known if this correlation is found in non-attached aggregates. In some infections, bacteria are trapped in highly viscous host material, e.g., CF sputum or wound slough, and we speculate that aggregates that are trapped in such material will likely not be able to disperse via regained flagellar motility.

These studies suggest that the dispersal of bacterial biofilm aggregates is a complex process which is influenced by multiple factors, although more data focusing specifically on biofilm dispersal of the aggregate phenotype are needed. The availability of nutrients, the presence of signaling molecules, and the mechanical forces upon the biofilm aggregates may all play a role in the dispersal of biofilm aggregates.

### The aggregate microenvironment

**Fig. 5:**
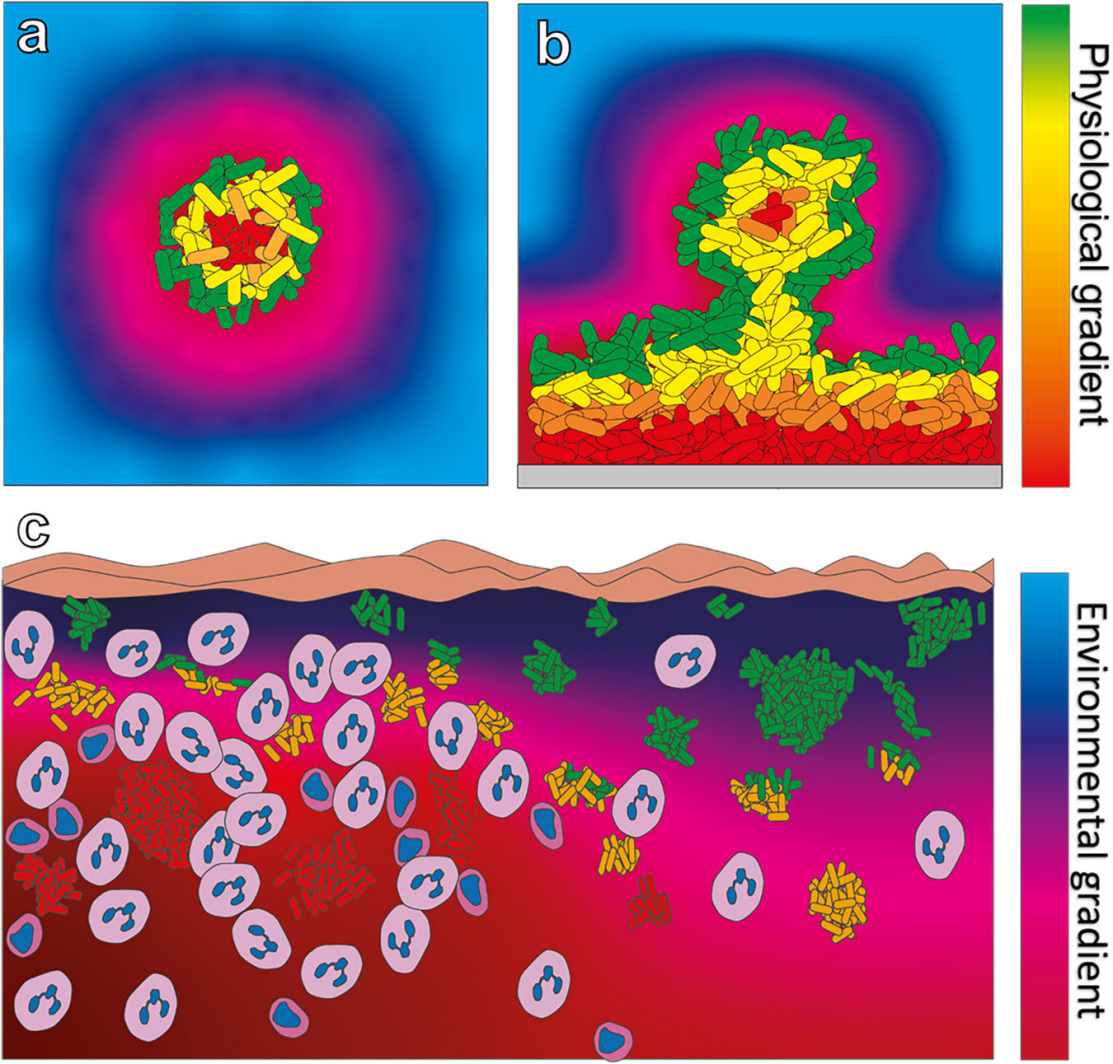
Gradient formation in biofilms. Green-red color scale depicts physiological gradients whereas red-blue color scale depicts an environmental gradient. **a** Non-attached aggregates will experience three dimensional gradient formation in the direction from their surface toward the center (or opposite e.g., for metabolic by-products). This can create a stratification in growth leading to different sub-populations. **b** For surface attached biofilms, the same diffusion phenomena will occur and will also depend on the three-dimensional structure. Again, sub-populations can form due to gradient formations. **c** Conceptual figure showing the influence of the immediate microenvironment on biofilm physiological state. Aggregates in the top-right side are close to the external environment and are distant from highly O_2_-consuming neutrophils. Aggregates in the lower-left are surrounded by neutrophils that creates an anoxic microenvironment.

The growth of individual aggregates is determined by environmental factors surrounding the aggregates (Fig. 5a). In viscous environments, bacteria will largely be constrained to their three dimensional position and thus, exchange of metabolic substrate and by-products will follow reaction-diffusion equations. These conditions have been thoroughly reviewed elsewhere^63–65^. But briefly, the occurrence of gradients in aggregates are determined by the reaction rate, diffusion characteristics and local boundary conditions. Example calculations are provided in Lichtenberg et al. (2022) that shows that even small aggregates can have internal hypoxic zones^65^. This has also been shown experimentally, where aggregates of *P. aeruginosa* with a radius of only 24 µm showed diminished internal O_2_ concentrations^66^. In the study by Wessel et al., gradients inside aggregates were thus seen even at fully oxygenated external conditions but in many environments the O_2_ concentration will not be at saturation. Hypoxic zones have, for example, been demonstrated in the mucus in lungs of people with cystic fibrosis (CF) as well as in the slough of chronic wounds^27,67^. Dynamics of the external environment influence the microenvironment of single aggregates and thus their boundary conditions. E.g., the recruitment and activation of PMNs in sputum from CF patients, will lead to local exhaustion of O_2_ due to production of their oxidative burst^68^. This releases reactive oxygen radicals that also follow reaction diffusion equation into aggregates^63^. The microenvironment surrounding aggregates is thus highly dynamic and their growth is not in the slightest restricted to the examples mentioned here but will most likely include several growth factors (e.g., carbon sources, electron acceptors, iron etc.), pH, and external stressors (e.g., antibiotics, ROS, secondary metabolites etc.). Examples of such dynamics has been shown for *S. aureus* where the transition to anaerobiosis was demonstrated by visualizing the localized expression of the lactate dehydrogenase gene via a ldh::gfp reporter strain^29^.

Non-attached aggregates and surface bound biofilms have the potential to experience similar microenvironments as both are limited by diffusion^64^. Bacteria inside simple flat biofilms that are attached to a solid surface, rely on diffusion from above the biofilm, while the surfaces of non-attached aggregates are more exposed in three dimensions. However, more complex diffusion patterns can occur in structured surface attached biofilms showing three-dimensional organization, such as flow cell mushroom structures (Fig. 5b). The same applies for the export of metabolic waste products. The gradient formation of metabolic products can lead to a heterogenic distribution of physiological states within the same biofilm^29,69,70^ which can lead to different subpopulations e.g., displaying different susceptibilities to antibiotics^71–73^. It is important to recognize the scale at which these things are discussed as emphasized by Kirketerp-Møller et al. that described the so-called Zone model that dictates that every single bacterium reacts to its own microenvironment^74^. In infections, bacterial physiology will thus be more complex and be determined by the immediate microenvironment which could be distinct even between neighboring aggregates (Fig. 5c).

### The ability of aggregates to tolerate antibiotics and evade immune responses

The increased tolerance toward antibiotic treatment and immune responses remains one of the defining features of surface attached biofilms^75^, and the available evidence suggest that these important traits are also common for non-attached aggregates.

For example, Alhede et al. found that *P. aeruginosa* aggregates grown in liquid media, exhibited tobramycin and colistin tolerance, as well as resilience towards neutrophils, comparable to that found for surface attached biofilms^33^. Similarly, non-attached aggregates of *S. aureus* have been shown to be highly tolerant towards both kanamycin, ciprofloxacin, erythromycin, and vancomycin^10^. Moreover, Pabst et al. demonstrated that gel-entrapped *S. aureus* aggregates were much more tolerant towards oxacillin, minocycline, and ciprofloxacin than planktonic cells^29^.

*P. aeruginosa* mutants that were deficient in the synthesis of Psl, Pel and alginate were shown to form aggregates in high density (0.8%) agar gels, and the bacteria in these aggregates were found to be more antibiotic tolerant than the corresponding strains growing dispersed in low density (0.2%) agar gels^28^. These results suggested that the biofilm matrix may not be necessary for the antibiotic tolerance of aggregates. However, subsequently it was reported that *P. aeruginosa mucA* aggregates (overproducing alginate) and *P. aeruginosa* Δ*wspF* aggregates (overproducing Pel and Psl) displayed increased tolerance towards tobramycin and ciprofloxacin compared to *P. aeruginosa* wild-type aggregates^76^. Recently, however, it was found that overproduction of exopolysaccharides confers a high metabolic burden on the bacteria, and results in subsequent low metabolic activity if the bacteria are situated in a nutrient/oxygen limited environment^77^. Thus, a difference in antibiotic tolerance between exopolysaccharide-overproducing aggregates and wild-type aggregates could be due to differences in the metabolic state of the bacteria. Accordingly, using an experimental setup where *P. aeruginosa* aggregates were cultivated under more homogeneous oxygen conditions than in the former study^78^, found that aggregates formed by alginate-overproducing mutants showed increased tolerance to tobramycin and meropenem, but not increased tolerance to ciprofloxacin. Furthermore, it was found that mutants that overproduce Pel or Psl did not display increased tolerance to tobramycin, meropenem and ciprofloxacin in comparison to wild-type aggregates^78,37–39^.

Since the first in-depth investigation of the phenotype of biofilms in chronic infections, the high local activity of inflammatory cells surrounding the biofilm aggregates has been apparent. However, aggregates and the inflammatory cells appear spatially separated, indicating that immune cells are unable to infiltrate the aggregates^79–81^ (Fig. 10a, b). The size of the aggregates appears to correlate with their ability to evade phagocytosis by innate immune cells. In liquid culture it was shown that aggregates with a diameter of more than 5 µm formed by *S. epidermidis, S. aureus, Escherichia coli, and P. aeruginosa* were less efficiently phagocytosed by neutrophils than smaller aggregates^82^. Similarly, it was shown that only surface attached aggregates with an area less than 50 µm^2^ (corresponding to a diameter of ~8 µm, assuming round aggregates) were efficiently cleared^83^. Thus, the size of aggregates appears to be a major determinant of the efficiency of clearance by immune cells. Besides the passive inhibition of phagocytosis due to size, *P. aeruginosa* in non-attached aggregates has been shown to actively kill PMNs through the secretion of the quorum sensing regulated rhamnolipid^33^. This biosurfactant has previously been attributed to surface-attached biofilms as the ones found in flow cells where it is produced in a density dependent quorum sensing-regulated manner^84–86^.

### Resemblance of non-attached aggregates to surface-attached biofilms

**Fig. 6:**
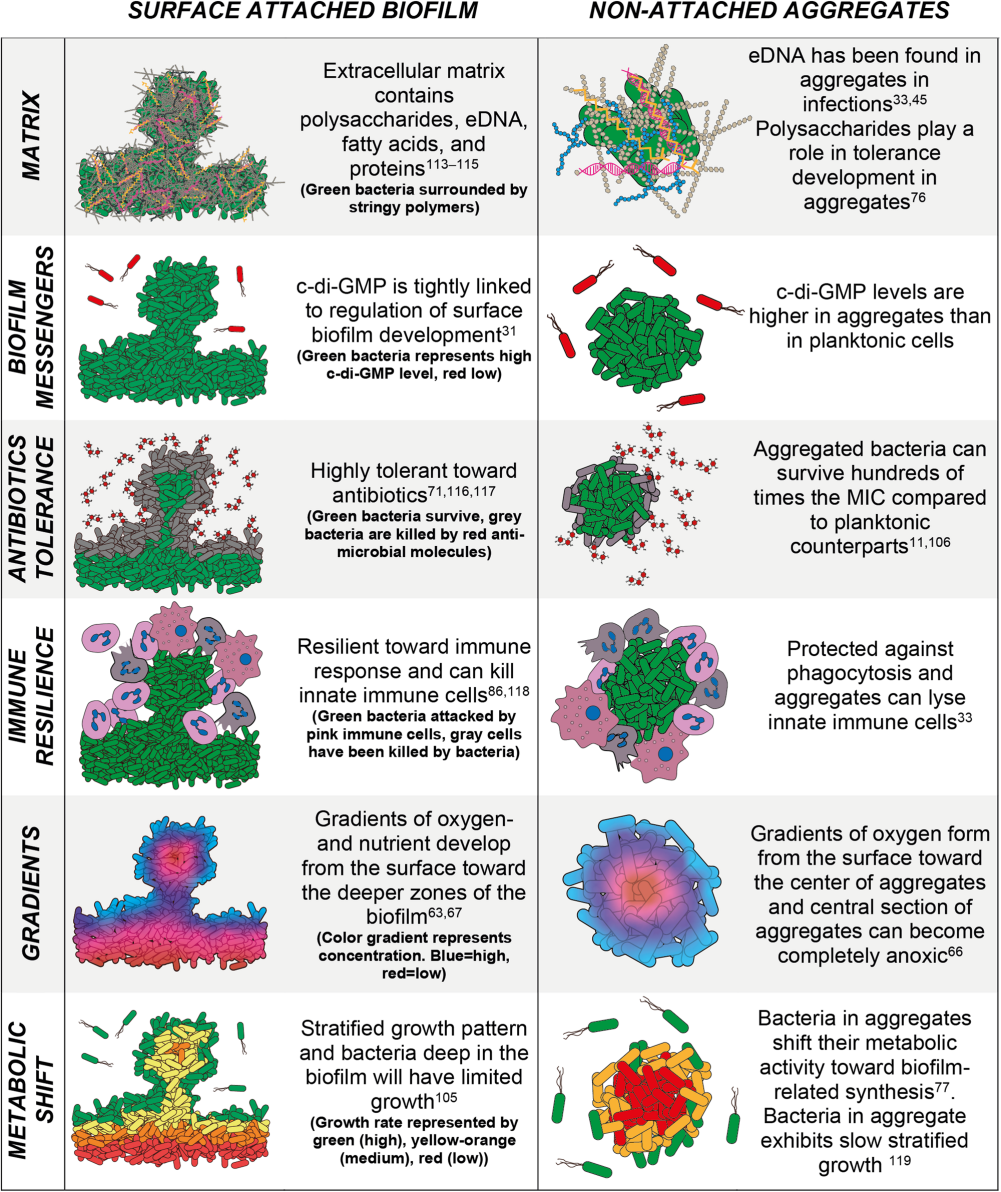
Phenotypic traits of surface attached biofilms and non-attached aggregates. Schematic comparison of phenotypic traits associated with to surface-attached biofilm and non-attached aggregates. *We have measured an elevated level of c-di-GMP in aggregates compared to planktonic cells, but these results are currently unpublished.

A central question is to what extent the bacteria in non-attached aggregates share phenotypic traits with the bacteria in surface-attached biofilms (Fig. 6)?

The consensus on what defines a biofilm is that it consists of a community of microbes living enclosed in extracellular matrix consisting of polysaccharides, proteins, lipids, and eDNA^13^. The matrix and stratified metabolic activity provide the microbes with an increased tolerance against antibiotics and other environmental stressors compared to planktonic cells and protect them against host immune responses^75^.

As discussed above, it is currently not clear whether the extracellular matrix is required for microbial aggregate formation in suspension and under conditions found at infectious sites^11,31,32,35^, as mechanisms of aggregate formation independent of bacterial matrix products also exists^28,30^. Although wild type aggregates and aggregates formed by mutants unable to produce matrix appear to be equally tolerant to antibiotics^28,30^, aggregates formed by mutants that overproduce matrix components appear to be more tolerant toward antibiotics than wild type aggregates^76^. The occurrence of stratified metabolic activity in microbial aggregates is not well explored but recent developments in single cell transcriptomics have suggested diverse physiological states within the same biofilm^29,69,70^ and between aggregates trapped in a secondary matrix^29,69,70,73^. Our knowledge from investigations of surface attached biofilms^87,88^ suggests that stratified metabolic activity occurs in aggregates despite their smaller physical dimensions.

Studies of surface associated *P. aeruginosa* biofilms have shown that the bacterial c-di-GMP level increases dramatically upon surface contact^55,89,90^. Using a fluorescent c-di-GMP reporter, based on a fusion between the c-di-GMP-regulated *cdrA* promoter and *gfp*^91^, we have found that the bacteria in agar-embedded *P. aeruginosa* aggregates have elevated levels of c-di-GMP compared to bacteria in planktonic culture. This is a strong indication that bacteria in non-attached aggregates share phenotypic traits with the bacteria in surface-attached biofilms, although these data are currently unpublished.

A low metabolic activity of the bacteria is a hallmark of biofilms and contributes substantially to the antibiotic tolerance displayed by biofilms^75^. Using a microcalorimetric approach, we have shown that c-di-GMP signaling is a major determinant of the metabolic activity of *P. aeruginosa* bacteria in planktonic culture, surface attached biofilms and aggregates^77^. The high c-di-GMP content of bacteria in biofilms forces them to rapidly spend a large amount of energy on the formation of extracellular matrix products, resulting in subsequent low metabolic activity. This suggests that the low metabolic activity of the bacteria in mature biofilms to some extent is a consequence of a c-di-GMP-regulated persistence strategy.

Although our knowledge on non-attached aggregates is still in its infancy, it appears that bacteria in aggregates share phenotypic traits with bacteria in surface-attached biofilms. Thus, our current knowledge suggests that aggregates should be regarded as a special form of biofilms that can be found across diverse environments.

### Selective pressures toward multicellular aggregation

Although the aggregate lifestyle provides numerous benefits, it will also limit the growth and mobility of the cells. The average growth rate for the whole population immobilized in a free floating aggregate is lower than for the same number of planktonic cells^33^. This is presumably due to the formation of chemical gradients from the surface of the aggregates towards the central regions as discussed above. The surface to volume ratio decreases with increasing aggregate size which will further restrict nutrient- and oxygen uptake, and decrease the potential growth compared to planktonic cells in in vitro cultures.

The biofilm phenotype is typically defined as sessile, and this is also partly true for non-attached aggregates. Cells in aggregates can of course passively be transported by mechanical forces, but an active movement is constrained by the aggregate matrix. If cells which constitute a non-attached aggregate exhibit the same transcriptomic switch as seen among cells in a surface attached biofilm, they will also down-regulate motility related genes. Thus, the movement of biofilm aggregates is largely determined by the local hydrodynamics due to liquid or mechanical forces such as ciliary movements.

In theory, aggregates are therefore not able to migrate to favorable niches during host colonization. However, the question is how frequent single cell migration is within the host. In the rare incidents where, single bacterial cells breach the barrier between skin, intestines or other commensally colonized compartments and enter sterile compartments of the body, it leads to a severe acute immune response^92^. The fitness trade-off between being organized as single cells or in biofilms is still widely debated and most likely does not have one single explanation. On one hand, single cell lifestyle is associated with fast growth and more even resource exploitation. On the other hand, the population is more vulnerable to environmental stressors where being organized in biofilm offers increased tolerance to many hostile conditions^93^. In infections, fast growing single cells may also, in very certain circumstances, lead to acute infections where pathogens such as *P. aeruginosa* or *Burkholderia cepacia* may migrate to the bloodstream causing bacteremia and possible death of the host within hours or days^92^. In other cases, the same organism can be found displaying slow growth in aggregates for decades which does not kill the host within hours, but years^92,94^. Most likely, the environment offers certain cues that bacteria respond to by either dispersing and being allowed to grow fast or by protecting themselves by encasement in biofilm where a rapid growth is then sacrificed.

Likewise, the environment may exert a selective pressure on attached vs. non-attached aggregation. In some infections, non-attached aggregates will be cleared very quickly e.g., in urethra and catheter infections a non-attached aggregate would be flushed away by flow. These are also the infections where surface attached biofilms are normally observed^95,96^.

### In vitro models for studying non-attached aggregates

**Fig. 7:**
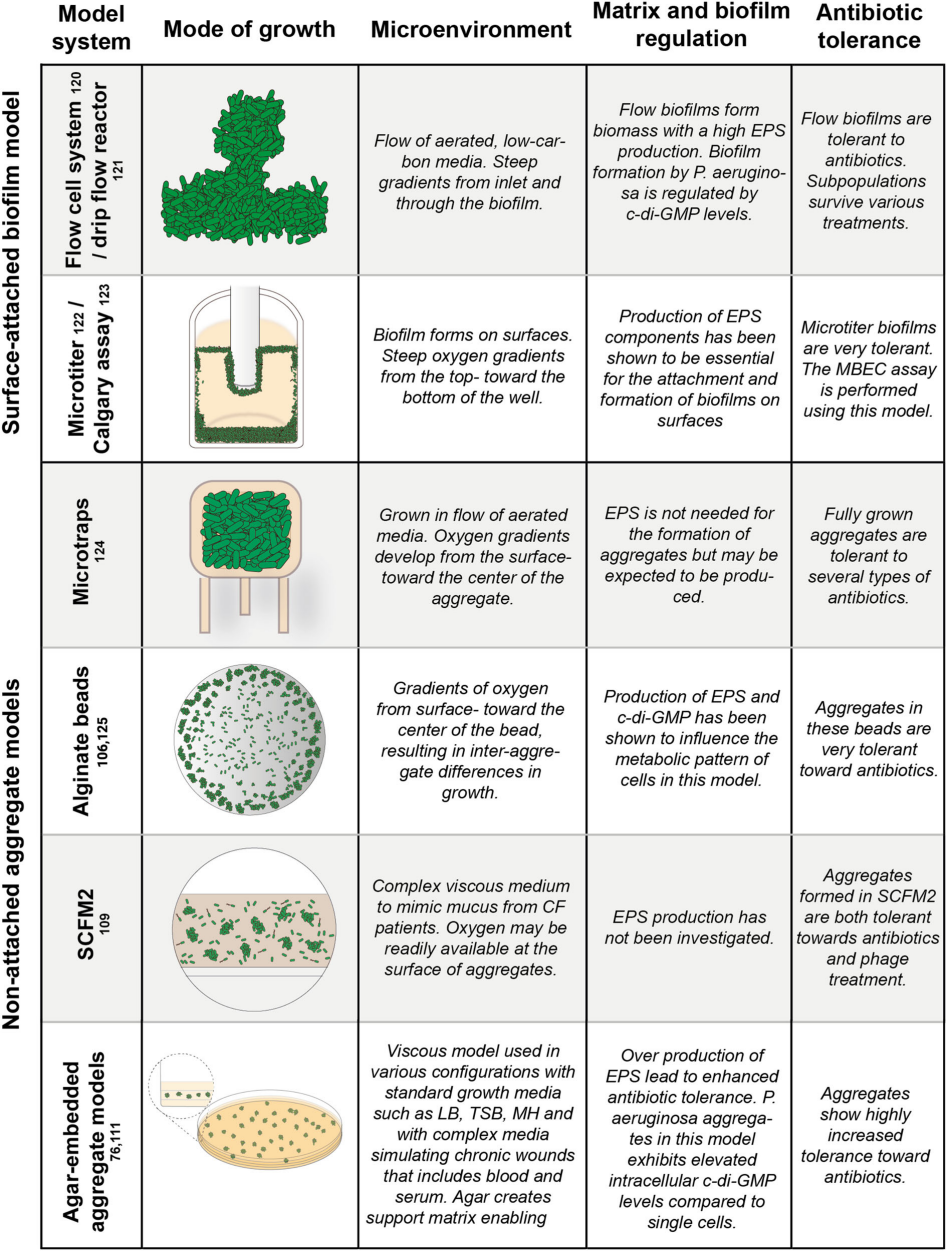
Examples of available in vitro biofilm models. Schematic breakdown of commonly used surface-attached biofilm models and model systems that aim to model non-attached aggregation. Common to all the models, is that host derived components and interactions is absent, except to some degree for SCFM2 where the chemical composition of CF sputum is mimicked.

The bulk of the current knowledge of biofilms has been gained using in vitro models where bacteria are grown on an innate surface as attached biofilms (Fig. 7). Systems such as the high-throughput microtiter biofilm assay have provided knowledge of the mechanisms of biofilm formation and antimicrobial tolerance and have enabled the screening of thousands of potential anti-biofilm compounds, whereas complex continuous flow cell biofilms have revealed structural mechanisms and regulatory pathways behind the development of surface biofilms^97–99^. Besides these ubiquitous biofilm models, CDC reactors, drip flow reactors, and most other in vitro biofilm models have the common trait of allowing planktonic cells to attach to an abiotic surface^100^. By providing these cells with liquid growth media the cells will grow into adherent biofilms. These biofilms can grow to be several hundred µm in thickness and often display structured architecture^98,101,102^.

Although perfect in vitro model systems rarely exist, an ideal non-attached aggregate in vitro model would aim at emulating in vivo/in situ phenotypic traits. Thus, emulation of the specific physicochemical microenvironment in question, as well as mimicking complex host-microbe interactions seem key in designing successful models.

One crucial aspect to consider, is the expression of extracellular polymeric substances (EPS) produced by biofilm cells but a clear understanding of the in vivo expression of EPS is still lacking. EPS play a critical role in the structure and function of in vitro biofilms, and their correct expression may be essential for accurately reproducing the behavior of biofilm aggregates in vivo where e.g., host derived carboxylates have been shown to impact biofilm development by CF pathogens^50^. In addition, the level of c-di-GMP which regulates biofilm formation and behavior is important. Finally, another essential factor to consider is antibiotic tolerance and resilience toward immune cells as found in non-attached aggregates^100,103^.

These characteristics are important for understanding how biofilm aggregates can persist in the presence of antimicrobial treatments and host defenses. Microenvironmental factors such as oxygen and nutrient gradients also play a critical role in the development and behavior of non-attached cells. Low oxygen levels can lead to increased tolerance of non-attached aggregates to antimicrobial treatments and also have an impact on the interactions between biofilm aggregates and host organisms^104,105^. Similarly, the availability of different nutrients can affect the growth and behavior of non-attached cells. To accurately mimic the in vivo non-attached phenotype, we first need to achieve a better understanding of the microenvironmental factors that are present in vivo, such as oxygen and nutrient gradients, as well as the correct production of EPS, level of c-di-GMP, antibiotic tolerance, and resilience towards immune cells. This will help in increasing our understanding of the complex interactions between microorganisms and their environment and ultimately aid in the development of more effective strategies for biofilm growth control in various environments.

Over the last decades, several model systems have been developed to model non-attached aggregates, primarily with a focus on understanding infectious biofilm aggregates (Fig. 7). Common to several of these model systems is that they use some form of immobilization of single cells with the aim of forcing the cells to propagate into dense aggregate. Wessel et al. used a gelatin-based three-dimensional printing strategy to trap a single cell in so-called microtraps^66^. These ~50 pL traps could be built on stilts in a flow system with a continuous flow of nutrient media surrounding it. The system was used to study the development of oxygen gradients through aggregates at various sizes. Although limited in clinical application due to the high level of surrounding oxygen and the absence of an immune component, this provided insights into the complex microenvironmental aspects of growth as a pseudo-suspended aggregate^66^.

Other groups have used low-viscosity gels to immobilize single cells. One example is the alginate bead model where aggregates develop from embedded single cells inside mm-sized beads that are formed in an alginate matrix^106,107^. An advantage with this model is the ability to form hundreds of beads with hundreds-thousands of individual biofilm aggregates inside^73^. As aggregates are growing, steep oxygen gradients develop from the edge of the bead towards the center. This creates a very stratified metabolic pattern between the various aggregates, with growth-limiting gradients going from the surface of the bead to the surface of each aggregate rather than gradients forming inside of each aggregate^73,106^. Aggregates grown in this model were shown to be highly tolerant towards common types of antibiotics, e.g., surviving 100x MIC of tobramycin^106^. Alginate can be found in abundance in the mucus surrounding biofilms in CF-associated lung infections, but in this model, it mainly serves as the immobilization matrix.

In an effort to capture in vivo chemical complexities, a synthetic CF medium was developed by Palmer et al. (SCFM)^108^ and further modified by Turner et al. (SCFM2)^109^ that closely resembles the viscosity and chemical composition of sputum from CF patients^108,109^. When single cells are seeded in this viscous medium comprised of amino acids, glucose, DNA, lipids, and mucins, these cells will form free-floating biofilm aggregates with a comparable size to that of aggregates found in CF-lung tissue^81,109,110^. In addition, *P. aeruginosa* aggregates formed in this model system have been shown to exhibit a comparable transcriptional profile as found in biofilms in CF infection, as well as exhibiting extensive tolerance towards both antibiotic and phage treatment^109,110^. Crone et al. developed a parallel model system that incorporated a chemically complex environment to model non-attached aggregates in a chronic wound model^111^. This model was based on growing aggregates in a sandwich of low viscosity agar media with wound-like components such as blood, serum, and animal derived media^111^. Both models were designed to mimic the highly nutrient-rich, but electron acceptor limited environments found in these infections.

More reductionistic model systems based on LB or minimal media with low percentage agar or agarose have provided insights into how EPS and the polymeric composition provided, can influence the development of antibiotic tolerance for cells in non-attached aggregates^76^. These simple models provide highly controlled conditions where essential phenotypic mechanisms can be investigated. A high degree of complexity will often compromise the time, effort, and cost of models and may not be necessary in all cases. Depending on the questions asked, more reductionistic approaches may be more optimal and tools for guiding such decisions have been developed^112^.

## Perspectives

With increased attention on non-attached aggregates, numerous questions arise. Do aggregates in infections resemble the ones studied in vitro? Are the mechanisms that operate during in vitro biofilm formation playing a role during aggregate formation in infections? Are the antibiotic tolerance mechanisms disclosed through in vitro biofilm studies relevant for aggregates in infections? Are aggregates in infections formed through recruitment or clonal growth? Does cooperation occur in aggregates, and if so, how do aggregated populations protect against cheating? To answer these questions, and many more, we need to bridge the gap between our in vitro systems and the environments found in the infections we want to mimic. Development of more relevant models should be guided by increased knowledge about infectious microenvironments.
